# Mitochondria–Lysosome Quality Flux in Aging Mesenchymal Stem/Stromal Cells: Quality-Control Bottlenecks and Therapeutic Opportunities for Regenerative Decline

**DOI:** 10.3390/cells15171548

**Published:** 2026-08-27

**Authors:** Lingling Cheng, Gaojie Song

**Affiliations:** 1 College of Veterinary Medicine, Gansu Agricultural University, Lanzhou 730070, China; vetcheng2025@163.com; 2 Jiangxi Provincial Key Laboratory of Cell Precision Therapy, School of Basic Medical Sciences, Jiujiang University, Jiujiang 332005, China

**Keywords:** mesenchymal stem/stromal cells, cellular senescence, mitochondrial quality control, mitophagy, mitochondria–lysosome quality flux

## Abstract

**Highlights:**

**What are the main findings?**

- The proposed mitochondria–lysosome quality flux (MLQF) framework reframes mitochondrial quality control in aging mesenchymal stem/stromal cells (MSCs) as a continuous process from damage recognition and cargo sorting to lysosomal delivery and terminal degradation.
- By separating direct MSC evidence from cross-model mechanisms and candidate pathways, MLQF identifies macroautophagy-dependent mitophagy as the best-supported route while defining the unresolved status of alternative mitochondrial cargo-clearance mechanisms.

**What are the implications of the main findings?**

- Delivery to an acidic compartment does not establish cargo degradation; rigorous MLQF assessment requires dynamic flux measurements, lysosomal blockade and MSC-relevant functional rescue.
- Localizing the rate-limiting MLQF step provides a testable basis for bottleneck-matched intervention and predicts when enhancing upstream clearance initiation will fail because the downstream lysosomal capacity remains limiting.

**Abstract:**

Aging mesenchymal stem/stromal cells (MSCs) lose regenerative capacity as redox imbalance, mitochondrial damage, defective organelle quality control and chronic inflammation converge. Yet these processes are commonly considered in isolation, obscuring whether damaged mitochondrial cargo reaches lysosomes and is ultimately degraded. Here, we define mitochondria–lysosome quality flux (MLQF) as an author-proposed, evidence-graded framework that tracks mitochondrial damage from recognition and sorting through lysosomal delivery to terminal lysosomal degradation in aging MSCs. The framework explicitly separates delivery to an acidic compartment from completed degradation and distinguishes direct MSC evidence from cross-model mechanisms and candidate pathways. MSC studies most strongly support macroautophagy-dependent mitophagy, particularly when assessed using dynamic flux reporters. By contrast, mitochondria-derived vesicles and microautophagy-like or piecemeal routes remain incompletely validated in MSCs. Studies in non-MSC systems further show that mitochondria–lysosome contact sites can support lysosomal acidification, although their contribution to natural MSC aging remains unresolved. By locating rate-limiting defects across this continuum, MLQF provides a testable basis for linking incomplete mitochondrial clearance to inflammatory signaling, lineage drift and regenerative decline, and for selecting bottleneck-matched interventions.

## 1. Introduction

Mesenchymal stem/stromal cells (MSCs) are a key reservoir of multipotent cells in adult tissues. They support the homeostatic maintenance and repair of bone, adipose and cartilage tissues, and have been widely explored in regenerative medicine and cell-based therapies [1]. With aging, MSCs progressively lose self-renewal capacity and lineage plasticity, changes linked to degenerative disorders including osteoporosis [2]. Experimental studies also connect MSC senescence to osteoarthritis-related phenotypes [3], while senescent MSC-derived extracellular vesicles show reduced protective capacity in osteoarthritis models [4]. Senescent MSCs typically show reduced proliferation, impaired clonogenicity, diminished osteogenic differentiation and a shift towards adipogenic commitment, accompanied by inflammatory remodeling of the secretome marked by upregulation of senescence-associated secretory phenotype (SASP)-like factors [2,5]. In passaging-induced senescence models of bone marrow-derived MSCs (BMSCs), senescent cells not only display cell-autonomous aging phenotypes but can also induce senescence features in neighboring cells through inflammatory paracrine signals, providing a cellular mechanism through which aging signals may be amplified at the tissue level [2,6]. The stability of MSC fate depends on tightly coordinated intracellular homeostasis. Mitochondria integrate energy production, redox balance and stress signaling, and are therefore essential for maintaining MSC stemness and lineage-fate decisions [7,8,9]. Direct evidence from natural aging models shows that BMSCs from aged mice and MSCs from older human donors commonly exhibit reduced mitochondrial membrane potential, impaired ATP production and a loss of osteogenic potential [5,10]. Recent intervention studies further suggest that restoring mitochondrial metabolism or mitochondrial homeostasis can improve MSC function and attenuate senescence-associated phenotypes, including through the regulation of glutamine metabolism, the enhancement of mitochondrial biogenesis and the induction of mitophagy [11,12,13,14].

Mitochondrial injury is not merely a correlate of MSC decline. Excess reactive oxygen species (ROS) can damage mitochondria, disrupt quality control and increase the cargo burden placed on lysosomes. Persistent mitochondrial damage may release components that activate cyclic GMP–AMP synthase (cGAS)–stimulator of interferon genes (STING) signaling and amplify inflammatory programs; however, the supporting literature spans general senescence mechanisms and MSC studies of mitochondrial structure rather than direct MSC-specific pathway validation [15,16,17]. Toxicant-induced BMSC senescence provides more direct, model-specific evidence for cGAS–STING activation under defined exposure conditions, but this finding should not be generalized to natural aging [18]. Mitophagy can limit this burden by removing damaged mitochondria and supporting MSC metabolism and function [19,20,21]. Whether mitophagy directly restrains mitochondrial DNA (mtDNA)-dependent inflammation in naturally aged MSCs remains unestablished. However, static measures such as microtubule-associated protein 1 light chain 3 (LC3)-II abundance, LC3 puncta, mitochondrial–LC3 colocalization or PTEN-induced kinase 1 (PINK1)/Parkin abundance cannot distinguish increased turnover from downstream blockade [8,19,22]. This limitation is more consequential if mitochondrial cargo can reach lysosomes through mitophagy, mitochondria-derived vesicles (MDVs) or microautophagy-like delivery. Dynamic assessment must therefore separate delivery to acidic compartments from terminal degradation. In BMSCs, combining pH-sensitive mito-Keima with lysosomal inhibition has linked impaired mitophagy flux to senescence and provided an applicable validation strategy [23].

MLQF does not replace established concepts of mitochondrial quality control, autophagic flux or mitophagy flux. Mitochondrial quality control encompasses a broader repertoire that includes mitochondrial dynamics, biogenesis, proteostasis and multiple degradation mechanisms [24]. Conventional autophagic flux assesses the formation and turnover of autophagic structures but is not necessarily mitochondrial cargo-specific [25], whereas mt-Keima and mito-QC primarily indicate the delivery of mitochondrial cargo to acidic compartments [26,27]. The distinct contribution of MLQF is to connect mitochondrial damage burden, route-specific cargo delivery, terminal lysosomal degradation and MSC functional outcome within a single evidence-graded analytical sequence. Here, MLQF decompensation refers to a state in which completed lysosomal clearance of damaged mitochondrial cargo becomes insufficient relative to the ongoing damage burden, whereas a bottleneck-matched intervention is selected according to the experimentally identified rate-limiting MLQF step. Nevertheless, MLQF remains an operational framework rather than a newly established molecular pathway or standardized quantitative index. Its application is limited by assay dependence, MSC heterogeneity, incomplete route-specific reporters and the difficulty of assigning terminal degradation to an individual delivery pathway.

To address this gap, we adapt multipathway concepts from mitochondrial quality control to MSC aging and define mitochondria–lysosome quality flux (MLQF) as an evidence-graded analytical framework [28,29]. Recent reviews have addressed MSC senescence and BMSC aging [30,31], mitochondria–lysosome contact sites (MLCS) in aging [32], and broader mitochondrial quality-control mechanisms [33]. However, these reviews have not organized MSC aging around a continuous, testable sequence linking mitochondrial damage input, cargo sorting, lysosomal delivery, terminal degradation and bottleneck-matched intervention. Rather than redescribing these themes or assigning MLCS the role of a newly discovered master hub, we use MLQF to determine whether damaged mitochondrial components progress from recognition and sorting to acidic-compartment delivery and terminal lysosomal degradation. We use MLQF decompensation to describe a state in which clearance completion declines while mitochondrial damage input remains high. This operational definition prioritizes dynamic clearance over isolated marker changes and is anchored in established flux assays and newer reporter-based approaches [22,26]. The framework is not intended to be exhaustive. It incorporates macroautophagy-type mitophagy, MDV-mediated delivery [27,34,35], and microautophagy-like or other piecemeal uptake [36,37]. These routes differ in cargo scale, machinery and strength of MSC-specific evidence. MLCS are considered candidate coordinating interfaces rather than the main source of novelty. Recent cross-model studies indicate that these contacts can contribute directly to lysosomal acidification [38,39], but whether this mechanism operates in naturally aged MSCs and becomes rate-limiting remains unresolved. Throughout, we distinguish direct MSC evidence, cross-model mechanistic evidence and candidate mechanisms. MLQF therefore offers a basis for locating bottlenecks and designing falsifiable experiments without assuming that every proposed route is active or limiting in aging MSCs.

## 2. MLQF: An Evidence-Graded Framework for Lysosome-Directed Mitochondrial Quality Control

MSC function depends on timely recognition and clearance of mitochondrial damage [40]. Within MLQF, mitophagy, MDVs and microautophagy-like or piecemeal delivery represent lysosome-directed execution routes, whereas MLCS may influence local membrane dynamics, metabolic signaling and terminal lysosomal function [38,39,41,42]. Evidence in MSCs is strongest for mitophagy and dynamic mitophagy-flux assessment [22,23,26,27]. MLCS have been associated with mitochondrial-network status and senescence phenotypes [43], whereas MDVs and microautophagy-like delivery remain supported mainly by non-MSC evidence [36,44,45].

Throughout this review, evidence is interpreted at three levels: direct MSC evidence linking pathway perturbation to dynamic flux and functional outcome; MSC-associated evidence based on static, correlative or incomplete flux measurements; and cross-model mechanistic evidence that primarily generates hypotheses for MSC validation. Findings in the third category are not considered established components of MSC aging unless they are reproduced in primary MSCs using cargo-specific flux measurements and MSC-relevant functional endpoints. This distinction is particularly important because tissue origin, donor characteristics, metabolic state and aging model may alter which MLQF step becomes rate-limiting.

### 2.1. Three Principal Lysosome-Directed Routes Within a Non-Exhaustive Classification

For analytical purposes, mitochondrial quality control can be organized into three principal lysosome-directed routes with different cargo scales and levels of evidence. Macroautophagy-type mitophagy sequesters whole mitochondria or large portions, MDVs transport locally budded components, and microautophagy-like or related piecemeal pathways internalize selected cargo through direct lysosomal engagement. This classification is not intended to exhaust all mitochondrial quality-control routes. Mitophagy has functional and flux-related support in MSCs; the other routes remain candidate mechanisms. Each route requires separate demonstration of cargo delivery to acidic compartments and completed degradation [36,44,45].

#### 2.1.1. Macroautophagy-Type Mitophagy: A Key Node Linking Differentiation Remodeling and Stress Clearance

In MSCs, mitophagy is better understood not as a passive disposal route for damaged components, but as a regulatory module that couples mitochondrial renewal, metabolic remodeling and cell-fate decisions [46]. Studies of osteogenic differentiation have shown that, in pathological settings such as hyperglycaemia, suppression of PINK1/dynamin-related protein 1 (Drp1)-related mitophagy is associated with impaired osteogenesis, suggesting that this axis contributes functionally to osteogenic output [47]. Consistently, stabilization of PINK1 or upregulation of Parkin can promote MSC osteogenic differentiation and new bone formation [46,48]. With respect to stemness maintenance, further studies have shown that mitophagy acts together with mitochondrial fission to support stemness-associated phenotypes and functions in bone marrow-derived MSCs [49]. In addition, because MSCs often reside in hypoxic niches or ischemic and inflammatory microenvironments, receptor-mediated mitophagy may provide a context-adaptive mode of regulation [50]. For example, under hypoxic conditions, receptor pathways involving BCL2-interacting protein 3 (BNIP3) are upregulated and are associated with maintenance of mitochondrial homeostasis and improved survival in MSCs. However, whether this pathway has a dominant role in the physiological hypoxic environment of bone marrow remains to be confirmed by further genetic evidence from primary bone marrow-derived MSCs [50].

A central methodological challenge in MSC mitophagy research is to avoid treating static markers as proxies for complete degradative flux [51]. Readouts such as LC3-II abundance, LC3 puncta or mitochondrial colocalization with LC3 reflect structural status or recruitment at a specific time point, but they cannot independently establish whether mitochondrial components have entered lysosomes and undergone successful degradation. Accumulation of autophagosomes or LC3-positive structures may reflect increased upstream formation, but it may also result from impaired downstream degradation caused by defective lysosomal acidification or fusion. Mitophagy flux should therefore be treated as a core endpoint [23]. In MSC systems, studies combining the lysosomal inhibitor bafilomycin A1 (BafA1) with the mito-Keima reporter system have established a quantitative approach for assessing mitophagy flux, providing a useful experimental paradigm for flux-based measurement in the MSC field [23]. In bone regeneration research, recent work has further shown that dynamic enhancement of mitophagy in bone marrow MSC-based tissue-engineering systems can improve cellular stress resistance and promote bone repair, supporting an operational link between mitophagy and functional phenotype from an application-oriented perspective [52].

MSC mitophagy studies should therefore prioritize flux over structural accumulation. Table 1 summarizes the evidentiary value and limitations of common assays, particularly the distinction between delivery to acidic compartments and completed lysosomal degradation. Component-selective maintenance may additionally involve MDVs, which are considered here as a candidate MLQF route.

Dynamic flux assays also have important technical and reproducibility constraints in MSC systems. Primary MSCs vary according to donor age, tissue source, passage number and culture conditions, all of which may influence basal mitochondrial and lysosomal activity [55,56]. Genetically encoded reporters can be affected by variable transduction efficiency, reporter expression and mitochondrial mass. Moreover, mt-Keima and mito-QC primarily indicate the delivery of mitochondrial cargo to acidic compartments and, when used alone, do not directly establish completed lysosomal proteolysis [26,27]. Lysosomal inhibitors are sensitive to dose and exposure duration and may themselves induce secondary mitochondrial or lysosomal stress. These challenges are amplified in vivo by the low abundance and heterogeneous identity of MSC populations, tissue autofluorescence and difficulties in achieving MSC-specific reporter expression. Reproducibility therefore requires matched sample processing, normalization to mitochondrial mass or reporter expression, predefined inhibitor conditions and time-resolved measurements. Reporter signals should also be validated orthogonally using lysosomal pH, proteolytic activity, mitochondrial cargo persistence and MSC-relevant functional endpoints [25].

#### 2.1.2. Mitochondria-Derived Vesicles as Candidate Routes for Component-Level Mitochondrial Quality Control

Unlike mitophagy, MDV flux has not yet been directly demonstrated in MSCs. This section therefore discusses MDVs as candidate execution routes rather than as validated mechanisms in MSCs. Mitochondria-derived vesicles represent a component-level quality-control strategy. Instead of engaging large-scale mitochondrial removal, cells can package selected damaged components into small vesicles through local mitochondrial budding and deliver them to the endosomal-lysosomal system for disposal [35,57]. This route may be particularly relevant to the sustained maintenance required under chronic oxidative stress. However, the core mechanistic evidence for MDVs currently comes mainly from non-MSC models. In mammalian cells, for example, factors such as Toll-interacting protein (TOLLIP) regulate Parkin-dependent trafficking of MDVs to lysosomes [57]. When canonical mitophagy is compromised, MDVs may also function as an alternative route for removing damaged mitochondrial components and maintaining organelle homeostasis [58].

Because MSCs in aging or inflamed tissues are often exposed to low-grade oxidative stress, MDVs may represent a candidate maintenance route for preserving mitochondrial component homeostasis in MSCs [59]. They are framed here as a working hypothesis that requires direct testing rather than as an established MSC mechanism. Future studies should move beyond the observation of vesicle formation and establish visualization and quantitative systems that track the delivery of defined mitochondrial cargo to lysosome-associated membrane glycoprotein 1 (LAMP1)-positive compartments in MSCs. Under genetic suppression of PINK1/Parkin or pharmacological blockade of lysosomes, it will also be important to determine whether delivery of defined mitochondrial cargo to LAMP1-positive compartments changes as a compensatory response. Establishing whether this pathway functions as an execution route within the MLQF network will require linking MDV turnover efficiency to MSC stemness maintenance, differentiation potential or senescence-associated phenotypes [44,60]. Mechanistically, MDVs emphasize selective partitioning and delivery. By contrast, microautophagy-like delivery represents a more direct mode of uptake into acidic compartments.

#### 2.1.3. Microautophagy-like and Piecemeal Pathways as Candidate Routes for Mitochondrial Component Recycling

Microautophagy-like pathways internalize cytoplasmic material through direct lysosomal or late-endosomal membrane remodeling without canonical autophagosome formation [36,61,62]. Cross-model studies have defined elements of their execution and selectivity and associated reduced endosomal microautophagy with age-related disruption of secretion and proteostasis [36,63,64]. A distinct 2024 study identified vesicles derived from the mitochondrial inner membrane (VDIMs), which form when inner-membrane material herniates through voltage-dependent anion channel 1 pores and is engulfed by nearby lysosomes with endosomal sorting complex required for transport (ESCRT) involvement [45]. Oxidative stress increased VDIM formation, supporting a role in selective removal of inner-membrane segments while sparing the remainder of the organelle [45]. These findings expand the cross-model repertoire of piecemeal mitochondrial quality control, but no direct MSC evidence yet shows VDIM formation or terminal degradation through this route.

In bone marrow-derived macrophages and RAW264.7 cells, ultrastructural and perturbation experiments showed that mitochondrial material can enter lysosomes through a microautophagy-like process [54]. The VDIM study provides an additional, mechanistically distinct example of lysosome-assisted piecemeal removal [45]. Testing either route in MSCs requires reporters that trace defined mitochondrial cargo into the lysosomal lumen, electron or three-dimensional microscopy, perturbation of route-specific machinery such as ESCRT components, lysosomal-blockade experiments and MSC functional endpoints [62,65]. Such evidence is needed to establish route identity, terminal degradation and biological relevance rather than cargo proximity alone.

Mitophagy, MDVs and microautophagy-like or piecemeal pathways converge on lysosomes but differ in cargo scale, delivery mechanism and MSC evidence (Table 2). This three-route organization is a practical, non-exhaustive classification; VDIM-mediated inner-membrane removal illustrates that additional route subtypes may emerge [45]. Defining their complementarity may reveal where clearance becomes rate limiting during aging. MLCS are consequently treated as candidate interfaces at which cargo sorting and delivery, organelle signaling and lysosomal function may intersect, not as validated master regulators.

These routes should not be regarded as completely independent or functionally equivalent. Mitophagy, mitochondria-derived vesicles and microautophagy-like pathways differ in cargo scale and execution machinery but converge on lysosomal processing and may share selected damage-recognition and endolysosomal-trafficking components. MDVs provide a selective route for transporting mitochondrial cargo to lysosomes that is distinct from wholesale mitochondrial turnover [35], while PINK1 and Parkin can participate in both canonical mitophagy and stress-induced MDV formation [66]. These relationships suggest partial functional overlap and potential compensation, although increased delivery through an alternative route would not overcome insufficient terminal lysosomal degradation. MLCS should not be considered an additional degradative pathway; rather, they may regulate mitochondrial fission and organelle dynamics through Rab7-dependent contact formation and resolution [41]. Thus, these pathways are mechanistically distinguishable but functionally interconnected. Their relative contributions and compensatory capacities in aged MSCs remain to be established directly.

**Figure 1:**
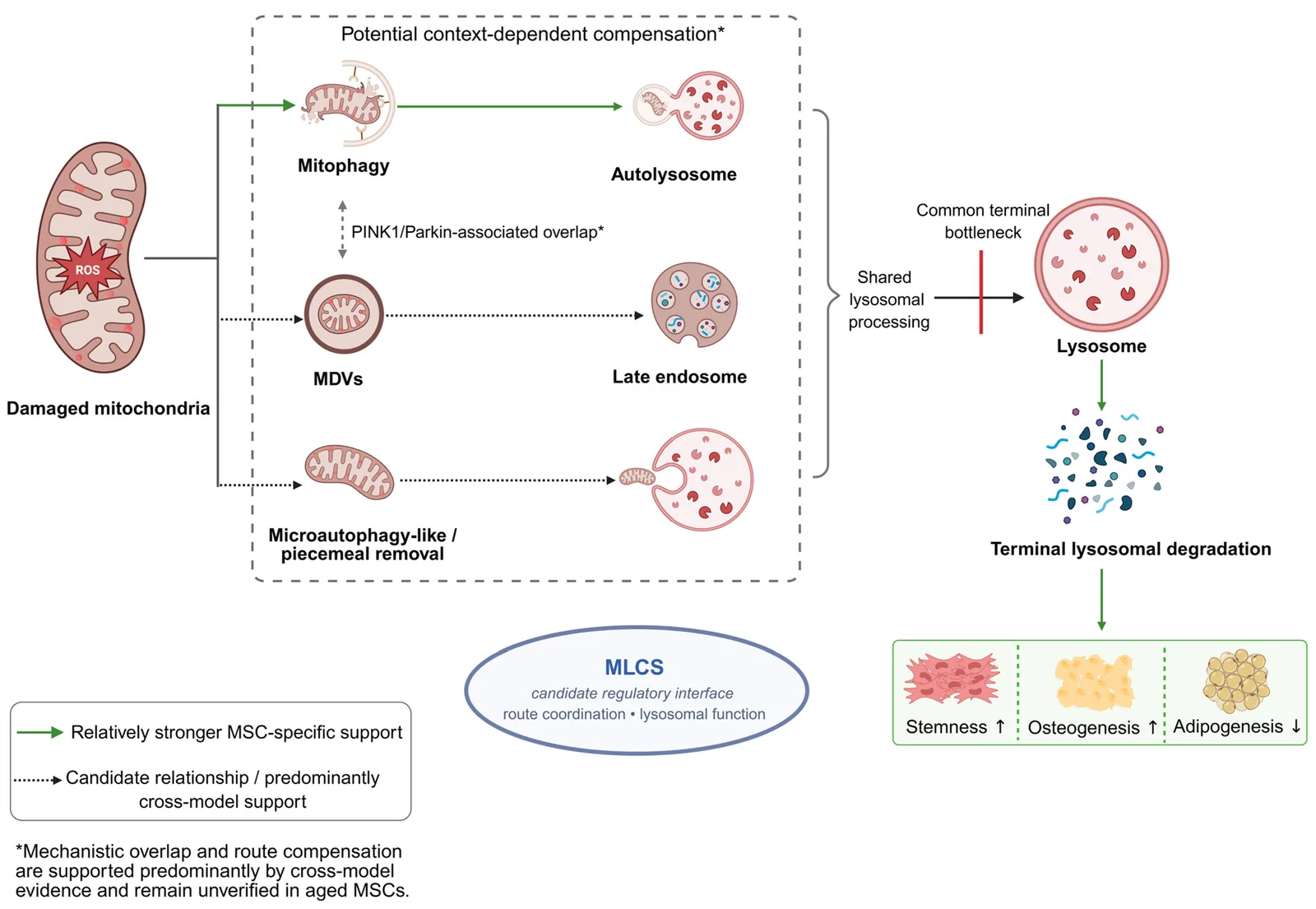
Evidence-graded architecture of the MLQF network in MSCs. The diagram depicts lysosome-directed mitochondrial quality control through macroautophagy-type mitophagy, mitochondria-derived vesicles (MDVs), and microautophagy-like or piecemeal removal. Mitophagy has comparatively stronger direct support in mesenchymal stem/stromal cells (MSCs), whereas MDVs and piecemeal routes remain candidate mechanisms supported mainly by non-MSC models. Mitochondria–lysosome contact sites (MLCS) are positioned schematically as candidate, context-dependent coordination interfaces; their placement does not imply that all three routes require MLCS. Delivery to an acidic compartment is distinguished from terminal lysosomal degradation. Functional benefits remain conditional on completion of degradation and require validation using dynamic flux assays. Dashed lines separate the three parallel functional outcomes; ↑/↓ denote direction of change.

Figure 1 integrates these routes within an evidence-graded MLQF architecture and depicts MLCS as candidate, context-dependent coordination interfaces rather than as an established coordinating hub in MSCs.

### 2.2. MLCS as Candidate Interfaces for Flux Coordination and Lysosomal Function

Mitochondria–lysosome contact sites are dynamic membrane microdomains that can support local membrane remodeling, ion exchange, metabolic adaptation and quality control in non-MSC systems [67,68,69]. In several human cell lines, the Rab7 GTPase cycle regulates contact formation and release and marks mitochondrial fission sites [41]. In primary sensory neurons, Charcot–Marie–Tooth disease type 2B (CMT2B)-associated RAB7A variants prolong contact dwell time and disrupt mitochondrial dynamics, showing that more or longer contacts do not necessarily indicate efficient clearance [70]. Transient receptor potential mucolipin 1 (TRPML1)-mediated lysosomal Ca^2+^ efflux can also couple lysosomal and mitochondrial Ca^2+^ homeostasis at contacts [71].

Direct MSC evidence remains limited. In the tunable matrix-stiffness model reported by Wang et al., mechanical cues altered MLCS abundance and mitochondrial-network status in association with MSC senescence phenotypes [43]. The study supports a possible role for contacts in adaptation to the mechanical microenvironment, but did not determine whether MLCS control cargo delivery into acidic compartments or terminal lysosomal degradation. At a downstream step, apoptotic vesicles from young MSCs restored autophagic flux in aged MSCs through Rab7-mediated autolysosome formation and improved bone mass and marrow adiposity in aged mice [72]. This provides MSC-based evidence that Rab7-dependent delivery and fusion are therapeutically tractable, but it is not direct evidence of MLCS manipulation.

Recent cross-model studies strengthen the link between MLCS and lysosomal function. In mammalian cell models, contact-dependent proton transfer from mitochondria promoted lysosomal acidification, and increasing organelle proximity enhanced acidification and proteolysis [38]. A complementary study spanning yeast and human cells linked age- or senescence-associated contact disruption to lysosomal de-acidification; an engineered inter-organelle linker restored acidification and autophagic flux and limited induction of major SASP factors in senescent human cells [39]. These findings establish contact-dependent acidification in non-MSC systems but not its rate-limiting role in naturally aged MSCs. Other studies connect MLCS to Parkin-dependent amino-acid homeostasis and transcription factor EB (TFEB) responses [73,74]. MSC studies should therefore pair live contact measurements with mitochondrial-cargo flux, lysosomal pH, proteolysis and functional endpoints.

### 2.3. Integrating Flux Regulation: From Isolated Pathways to a Systems-Level View of MSC Aging

MLQF decompensation, as defined in this Review, does not refer to a change in any single static marker. It describes a reproducible mismatch between mitochondrial damage burden, lysosome-directed delivery of damaged mitochondrial components and completion of terminal lysosomal degradation. This distinction is important because LC3-II abundance, PINK1/Parkin expression and organelle colocalization can indicate pathway engagement but cannot, on their own, establish that mitochondrial cargo has been cleared. MLQF assessment should therefore ask whether damaged mitochondria or mitochondria-derived components are recognized when needed, routed to acidic compartments and degraded in a lysosome-dependent manner. We interpret this process across four linked dimensions: damage input, mitochondrial cargo delivery, terminal degradative capacity and blockade-based validation (Table 3). Each dimension has a defined evidentiary boundary, and MLQF decompensation should be inferred only when these readouts are considered together rather than from any single marker.

MLQF decompensation is therefore best understood as a state in which increased damage burden coexists with insufficient terminal degradative output. In practice, young or healthy reference MSCs should be processed in parallel with aged or stressed MSCs to minimize batch effects. Mitochondrial damage input should first be confirmed to be increased. pH-sensitive mitochondrial reporter systems should then be used to assess delivery of mitochondrial cargo to acidic compartments. Terminal degradation should be evaluated from lysosomal-blockade-induced changes in cargo persistence together with lysosomal pH or proteolytic activity [22,23]. Complementary approaches, including mito-QC, electron microscopy with immunolabeling and colocalization with acidic compartments, can improve reproducibility and cross-model comparability [26,27,51]. This principle is particularly important in MSC aging studies, because autophagosome accumulation, PINK1/Parkin upregulation or increased organelle contacts in senescent cells may reflect enhanced compensatory initiation, but may also result from impaired terminal degradation.

Integrating mitophagy, MDVs, microautophagy-like or piecemeal delivery and MLCS reframes MSC aging as a mismatch across the mitochondrial quality-control network rather than failure of a single pathway [75,76]. MSC evidence links mitophagy to osteogenic output [46,48], MLCS abundance to mitochondrial status and senescence phenotypes [43], and mitochondrial decline to lysosomal abnormalities [77]. Reduced acidification and proteolytic capacity are plausible downstream bottlenecks [78,79], while non-MSC studies directly support contact-dependent lysosomal acidification [38,39]. Whether this mechanism controls mitochondrial-cargo degradation or becomes rate limiting in naturally aged MSCs remains unknown. The available evidence therefore supports a multilayered model of damage overload, impaired coordination and insufficient lysosomal capacity rather than failure of a universal hub.

This framework changes intervention logic from indiscriminate pathway activation to bottleneck identification. Stabilizing PINK1/Parkin could enhance upstream damage labeling, but will not restore output if acidification, fusion or hydrolysis remains limiting [79]. This conditional relationship requires direct testing in MSCs. Section 3 therefore uses MLQF to distinguish defects in recognition, delivery coordination and terminal degradation.

**Table 3:** Operational criteria for resolving MLQF decompensation in aging MSCs.

Dimension	Core Question	Representative Readouts	Key Interpretative Boundary	References
Damage input	Is mitochondrial damage input increased relative to matched young or healthy MSCs?	Mitochondrial membrane potential; mitochondrial ROS; mtDNA damage; mitochondrial fragmentation	Defines damage burden, not delivery or degradation	[5,10,23]
Lysosomal delivery	Does damaged mitochondrial cargo reach acidic lysosomal compartments?	mito-Keima or mito-QC, interpreted with lysosomal blockade where applicable	Demonstrates delivery to an acidic compartment; does not establish terminal proteolysis	[22,23,26]
Terminal degradation	Is mitochondrial cargo proteolytically cleared after lysosomal entry?	Lysosomal pH; cathepsin activity; DQ-BSA; mitochondrial cargo persistence or loss	Distinguishes completed degradation from cargo accumulation	[22,27,51,78,80]
Flux validation	Does lysosomal blockade reveal ongoing cargo turnover rather than static accumulation?	BafA1 or chloroquine with cargo-specific accumulation and LC3/p62 interpretation	Valid only when paired with mitochondrial cargo-specific readouts	[22,23,51]

## 3. Systemic Mismatch in the Quality-Flux Network: Key Nodes Associated with MSC Aging

Section 3 applies the MLQF criteria to potential rate-limiting steps in aged MSCs. The focus is whether rising mitochondrial damage remains matched by recognition, inter-organelle coordination and lysosomal degradation. Failure at any step may shift the network from compensation to decompensation and contribute to inflammatory amplification, lineage drift and functional decline.

### 3.1. Damage-Input Overload and Impaired Recognition: Initiating Failures in the Flux Network

Under aging or prolonged stress conditions, MSCs often exhibit impaired redox homeostasis, persistent inflammatory stimulation and delayed metabolic adaptation. This state is accompanied by increased generation and accumulation of damaged mitochondrial components, which in turn raises the demand on mitochondrial quality-control and clearance pathways [2]. Hyperglycaemic stimulation can induce mitochondrial dysfunction and senescence-associated phenotypes in synovial MSCs, suggesting that metabolic abnormalities may directly increase mitochondrial damage burden and trigger senescence programs [81]. In bone marrow-derived MSCs, reduced mitochondrial membrane potential, heightened oxidative stress and restricted mitophagy flux often occur together with reduced proliferative capacity and impaired osteogenic function [5,23]. Abnormalities in the mitochondrial respiratory chain, such as suppression of *Ndufs6*, have also been associated with senescence in adult MSCs [82].

The conversion of damage signals into clearance signals may also become less efficient. The PINK1-Parkin axis is one of the most frequently examined entry points in this process [2,78]. In bone marrow MSCs, environmental toxic stress can suppress mitophagy flux and aggravate senescence and functional impairment. This interpretation is supported by flux validation using the mito-Keima reporter system under lysosomal inhibition [23]. In inflammatory cytokine-induced stress models, Parkin-mediated mitophagy can buffer tumor necrosis factor-α (TNF-α)-associated damage and help maintain cellular homeostasis [83]. Beyond exogenous stress, endogenous inhibitory circuits within MSCs may also limit this entry step. Leucine-rich repeat-containing protein 17 (LRRc17) suppresses mitophagy through signaling involving mechanistic target of rapamycin (mTOR) and phosphoinositide 3-kinase (PI3K) and promotes senescence-associated phenotypes in bone marrow MSCs, indicating that the recognition and labeling step itself may be subject to endogenous negative regulation and may amplify flux defects when damage input persists [84].

As discussed in Section 2.1.1, static markers alone cannot determine whether damaged mitochondrial components enter lysosomes and complete degradation. Evaluation of this step should therefore remain centered on terminal degradative events. This includes flux measurements under lysosomal inhibition, validation with dedicated reporter systems and, where feasible, the incorporation of quantitative tools to improve comparability and reproducibility across experimental settings [22,51,85].

### 3.2. Disrupted MLCS Coordination and Functional Dysregulation

Clearance requires coordinated positioning of mitochondria, lysosomes and degradative machinery, but current MSC data do not establish MLCS as the causal center of this process. Matrix stiffness alters MLCS dynamics and mitochondrial architecture in association with BMSC senescence phenotypes, identifying contacts as a potentially manipulable node [43]. Separately, Rab7-dependent restoration of autolysosome formation reverses senescence-associated phenotypes and alleviates bone loss in aged models [72]. The latter supports Rab7-dependent delivery and fusion, but not a causal effect of abnormal MLCS dynamics on MSC aging. Existing studies therefore implicate contact dynamics and downstream autolysosome formation at different levels of the clearance chain, without yet connecting them in the same causal flux experiment.

Mechanistic interpretation of these MSC observations still depends largely on non-MSC systems. MLCS are dynamic membrane microdomains that can facilitate metabolite and ion exchange and coordinate membrane remodeling with downstream clearance events [67]. In human cell lines, the GTP-binding and hydrolysis cycle of Rab7 regulates contact formation and dissociation and helps position mitochondrial fission sites [41]. Fis1-associated complexes also influence spatial coupling between lysosomal and mitochondrial networks [86]. In sensory neurons, CMT2B-associated RAB7A variants prolong contact dwell time while disrupting mitochondrial dynamics, showing that an increase in contact duration does not necessarily indicate more efficient clearance [70].

Cross-model evidence also links MLCS to signaling and lysosomal function. TRPML1-dependent lysosomal Ca^2+^ release transfers Ca^2+^ to mitochondria in neuronal models and patient fibroblasts [71]. Split-GFP-based sensors show that α-synuclein can perturb contacts and TFEB translocation [74], and optogenetic contact manipulation can induce mitochondrial fission in HeLa cells [87]. Parkin also participates in amino-acid homeostasis at contact sites [73]. More directly, contact-dependent proton transfer supports lysosomal acidification, whereas age- or senescence-associated contact disruption reduces acidification and autophagic flux [38,39]. Whether these mechanisms operate in aging MSCs remains unknown.

Therefore, MSC studies should quantify the frequency, dwell time and spatial distribution of contact events under live-cell conditions, and validate these measurements in parallel with mitochondrial fission, mitophagy flux and terminal lysosomal degradative capacity. Existing imaging and analytical workflows can provide methodological support for this direction [68]. Interpretation should be anchored to whether damaged mitochondrial components enter acidic compartments and complete degradation, rather than assuming that increased contacts necessarily indicate enhanced clearance. This principle is consistent with established approaches for autophagic flux analysis [22].

### 3.3. Insufficient Terminal Degradation: Lysosomal Dysfunction and Defective Transcriptional Adaptation

Within the MLQF framework, terminal lysosomal degradative capacity may become a rate-limiting node that determines whether the system can complete effective turnover. Even when damaged mitochondria have been recognized and delivered to lysosomal compartments, insufficient acidification, hydrolysis, fusion or membrane stability can cause damaged mitochondrial components to persist, generating an ineffective flux state in which initiation is increased but degradation remains blocked [77,78]. Thus, the key issue in MSC aging is not only whether upstream mitophagy is activated, but whether damaged mitochondria and their derived components truly enter acidic compartments and undergo terminal degradation.

Available MSC studies provide convergent, but not yet definitive, support for this view. In a serial-passaging model of senescence in human umbilical cord MSCs, reduced cell viability occurs alongside concurrent impairment of mitochondrial and lysosomal function, suggesting that increased mitochondrial damage burden and reduced lysosomal degradative capacity may overlap during the aging process [77]. A mechanistic review further emphasizes impaired lysosomal acidification, reduced proteolytic activity and restricted autophagic substrate turnover as the key features of MSC aging [78]. More relevant from an intervention perspective, pharmacological activation of the TFEB-mediated autophagy-lysosome gene program in bone marrow-derived MSCs and bone-aging models improves autolysosomal function, attenuates senescence-associated phenotypes and reduces bone loss [80]. This evidence points to a focused interpretation: enhancing lysosomal biogenesis and degradative capacity may be closer to restoring MLQF output than simply increasing upstream autophagy initiation.

Lysosomal degradation bottlenecks are also reflected in impaired autophagosome-lysosome fusion and insufficient autolysosome formation. A substantial part of the relevant molecular evidence currently comes from non-MSC systems, including studies of the syntaxin 17 (STX17)/synaptosomal-associated protein 29 (SNAP29)/vesicle-associated membrane protein 8 (VAMP8) fusion complex, stress-signal regulation of terminal fusion and other membrane-dynamics processes that affect the closure of the autophagic flux cycle [88,89]. These studies provide mechanistic reference points for understanding potential fusion bottlenecks in MSCs, but direct validation through dynamic flux readouts in primary MSCs remains necessary. By contrast, studies of human bone marrow-derived MSCs in the context of osteoporosis have provided more MSC-specific clues. Impairment of the APPL1-Myoferlin axis can induce lysosomal stress, inhibit autophagosome degradation, promote autophagosome accumulation, reduce autolysosome formation and drive adipogenic lineage drift [85]. This finding associates defective autophagosome degradation with adipogenic lineage drift; whether mitochondrial cargo clearance is directly impaired remains to be tested. Similarly, apoptotic vesicles derived from young bone marrow MSCs can be taken up by senescent MSCs and restore autolysosome formation and autophagic flux by supplementing Rab7, thereby reversing senescence-associated phenotypes and alleviating bone loss in aged mice [72]. This finding reinforces the Rab7-autolysosome axis as an actionable component of terminal degradation, but it should not be interpreted as direct manipulation of MLCS.

Lysosomal pH maintenance and membrane stability also shape terminal degradative capacity. Direct BMSC evidence supports the functional importance of lysosomal ion homeostasis: during osteoblast differentiation of bone chip-derived BMSCs, TMEM175 expression increased, whereas TMEM175 knockdown or pharmacological inhibition with 4-aminopyridine impaired lysosomal function and autophagic clearance and reduced matrix mineralization and osteogenic-marker expression [90]. Although this study did not examine aged MSCs or mitochondrial cargo-specific flux, it links terminal lysosomal competence to osteogenic output in an MSC-relevant system. In mouse embryonic fibroblasts and human cell lines, mechanistic target of rapamycin complex 1 (mTORC1) controls lysosomal catabolic activity through vacuolar H+-ATPase (V-ATPase) assembly [91,92]. Recent studies add contact-dependent proton transfer from mitochondria as a complementary acidification mechanism and link its disruption to aging-associated loss of autophagic flux in non-MSC systems [38,39]. Endosomal sorting complex required for transport (ESCRT)-dependent membrane repair helps maintain lysosomal integrity [93], whereas lipofuscin can increase lysosomal membrane permeability in retinal pigment epithelial and related mouse models [94]. Together, these findings suggest that terminal failure may reflect combined defects in acidification, hydrolysis, fusion, membrane repair and transcriptional adaptation. Whether V-ATPase regulation, contact-dependent acidification or another component is rate limiting in aged MSCs requires direct comparison using mitochondrial flux reporters, lysosomal pH and proteolysis assays, and osteogenic or adipogenic endpoints.

### 3.4. Vicious Cycles and Phenotypic Outputs: Flux Mismatch and MSC Aging

When damage input, delivery coordination and terminal lysosomal degradation are concurrently impaired, MSCs no longer merely accumulate stochastic damage. Instead, they may enter a state of MLQF decompensation. Disruption of mitochondrial homeostasis and insufficient autophagy-lysosome turnover may together favor the accumulation of undegraded damaged mitochondrial components. At the same time, amplified inflammatory transcriptional programs may link organelle damage to senescence-associated signals at the cellular and tissue levels. In bone marrow-derived MSCs from middle-aged mice and corresponding bone-aging models, pharmacological enhancement of TFEB-mediated autophagy and lysosomal gene programs improves MSC phenotypes and alleviates bone loss, supporting lysosomal degradative capacity as a candidate intervention node [80].

Cytosolic DNA sensing is a plausible link between mitochondrial damage and inflammation. Yes-associated protein (YAP)/transcriptional coactivator with PDZ-binding motif (TAZ) signaling modulates stromal aging through cGAS–STING signaling in vivo [95], and metabolic stress couples mitochondrial damage, cytosolic mtDNA and cGAS–STING activation in pancreatic β cells [96]. Mito-QC reporter mice and human-cell studies further support mitophagy-dependent restraint of mtDNA-driven cGAS–STING inflammation [97]. Whether this pathway dominates SASP induction in naturally aged BMSCs remains untested and requires primary-cell time courses, MSC-specific perturbation and flux measurements [53,97]. Interleukin-6 (IL-6) and TNF-α may in turn increase oxidative stress through nuclear factor κB (NF-κB)-related signaling [6,83], but the strength of this feedback in aged BMSCs remains to be defined.

Flux restriction may also alter lineage output. PINK1 stabilization and mitophagy enhancement promote osteogenesis and bone formation in experimental models [46], and mitophagy or broader mitochondrial quality control has been linked to BMSC skeletal aging, osteoporosis and fracture repair [98,99]. Nicotinamide adenine dinucleotide (NAD^+^) depletion impairs mitochondrial metabolism, osteogenic signaling and bone repair [100], whereas targeting inducible nitric oxide synthase (NOS2) improves mitochondrial function and osteogenesis in aged human BMSCs [101]. Elevated mitochondrial ROS is associated with adipogenic drift [102]. Repetitive-element RNA sensing [103] and DNA-methylation–autophagy pathways [104,105,106] may add inflammatory and epigenetic layers, but should not be assigned directly to MLQF without flux evidence. Overall, the evidence supports a working model in which mismatch among damage input, delivery and degradation may contribute to inflammation, lineage drift and regenerative decline. Stratifying these defects could guide sequential or combined control of damage and lysosomal capacity [22,80,97].

**Figure 2:**
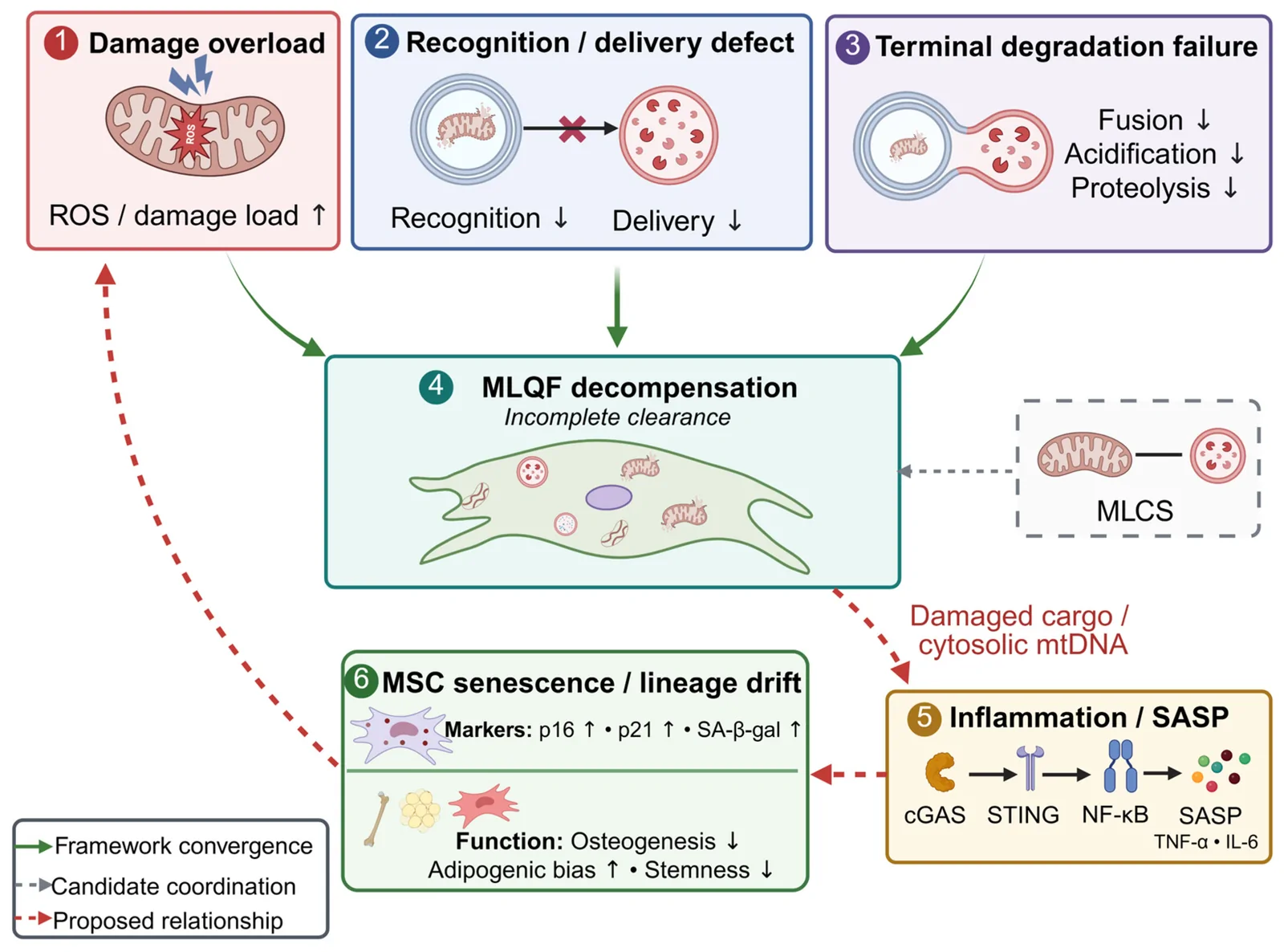
Proposed vicious-cycle model linking MLQF decompensation to MSC aging phenotypes. Mitochondrial damage overload, impaired cargo recognition or delivery, and terminal lysosomal degradation failure may independently or jointly converge on MLQF decompensation, characterized by incomplete clearance of damaged mitochondrial cargo. Mitochondria–lysosome contact sites (MLCS) are shown as a context-dependent candidate coordination interface rather than an established causal node. Incomplete clearance may promote the persistence of damaged mitochondrial cargo and cytosolic mitochondrial DNA (mtDNA), thereby activating cyclic GMP–AMP synthase–stimulator of interferon genes (cGAS–STING) and nuclear factor κB (NF-κB) signaling and enhancing the senescence-associated secretory phenotype (SASP). These inflammatory signals may reinforce MSC senescence and lineage drift, characterized by increased p16 and p21 expression and SA-β-gal activity, reduced osteogenesis and stemness, and increased adipogenic bias. Senescent MSC phenotypes may, in turn, further increase mitochondrial stress and damage load. Green solid arrows indicate convergence on the MLQF framework, gray dotted arrows indicate candidate coordination, and red dashed arrows indicate proposed relationships that have not been established as a complete causal circuit in naturally aged MSCs. ↑/↓ denote direction of change; × indicates impaired or blocked recognition/delivery.

Figure 2 summarizes this candidate vicious cycle of damage overload, restricted delivery, insufficient degradation, inflammatory feedback and lineage drift.

## 4. Targeting the MLQF Network: Mechanism-Guided Interventions for MSC Aging

Intervention should begin by locating the dominant bottleneck—damage input, delivery coordination, terminal degradation or a mixed defect—rather than by broadly activating autophagy or antioxidant pathways. Table 4 therefore organizes representative strategies into upstream correction, MLCS-associated coordination, terminal-capacity enhancement and multi-node regulation, while distinguishing direct MSC evidence from skeletal and cross-model support. Restoration should be judged by dynamic flux together with MSC functional endpoints.

### 4.1. Correcting Upstream Damage Sensing: Reducing Damage Input and Restoring Recognition Thresholds

For MLQF defects dominated by input overload or blunted recognition, intervention should not stop at increasing autophagy markers. Instead, it should aim to restore the match between damage signals and clearance responses. Upstream correction can proceed through two routes. The first is to reduce the generation of new mitochondrial damage and thereby lower the burden on downstream clearance. The second is to improve energy sensing, which may facilitate damage recognition and autophagy initiation. On this basis, NAD^+^ supplementation and AMP-activated protein kinase (AMPK) activation currently represent two relatively actionable upstream strategies. However, they act at different levels of the flux hierarchy. The former is more closely aligned with mitochondrial antioxidant defense and restoration of metabolic homeostasis, whereas the latter mainly acts on energy sensing and autophagy initiation.

Declining NAD^+^ weakens sirtuin (SIRT) family-dependent deacetylation and is associated with reductions in bone marrow stromal and osteoprogenitor cells, as well as bone loss [120,121,122]. In BMSCs, NAD^+^ loss is associated with impaired oxidative phosphorylation, osteogenesis and fracture repair [100]. In aged mice, NMN treatment promotes MSC expansion and shifts lineage output towards osteogenesis through SIRT1-associated mechanisms [107]. Recent work in an excess-glucose model showed that disruption of the NAD^+^/SIRT1 axis rapidly reduced mitochondrial activity in MSCs and biased lineage commitment away from osteogenesis, whereas NMN supplementation restored mitochondrial activity and osteogenic commitment in a SIRT1-dependent manner [123]. Together with evidence linking NAD^+^ decline to impaired MSC osteogenesis and reduced bone-repair capacity, these findings support an upstream role for NAD^+^/SIRT signaling in metabolic and lineage-fate correction, rather than direct restoration of terminal degradative flux [100,122]. The evidence for SIRT3 does not map entirely onto the same mechanistic layer. SIRT3-mediated deacetylation of mitochondrial antioxidant systems, including superoxide dismutase 2/manganese superoxide dismutase (SOD2/MnSOD), mainly supports the maintenance of redox homeostasis [124,125]. By contrast, a direct link among SIRT3, mitophagy, BMSC aging and bone loss is supported primarily by the study by Guo et al. [126]. Therefore, the NAD^+^/SIRT axis is better positioned as an upstream corrective strategy for input-overload-associated MSC aging, rather than being directly equated with restoration of terminal degradative flux. Whether this axis broadly restores the terminal degradative output of damaged mitochondrial components still needs to be tested using mito-Keima, mito-QC and comparisons before and after BafA1 treatment.

AMPK activation offers a second upstream entry point. As an energy-stress sensor, AMPK couples metabolic stress to autophagy initiation, in part through unc-51-like kinase 1 (ULK1)-dependent mechanisms [109,127]. Recent MSC evidence places this pathway in a functional context: activation of the AMPK–ULK1–autophagy axis has been linked to osteogenic differentiation and MSC-based bone regeneration [128]. In MSC studies, 5-aminoimidazole-4-carboxamide ribonucleoside (AICAR) has been shown in specific models to alleviate senescence-associated phenotypes and improve proliferative or functional readouts [110]. Metformin reduces DNA damage and senescence markers in models of experimentally induced senescence and chronic kidney disease-associated injury, and part of its effect is linked to AMPK-dependent enhancement of autophagy [111,112]. However, most of these studies remain at the level of general autophagy markers or functional phenotypes and do not yet demonstrate that AMPK activation is sufficient to restore mitophagy flux in MSCs. Future studies should directly quantify the efficiency with which damaged mitochondrial components enter acidic compartments and complete terminal degradation. These flux readouts should then be analyzed together with MSC functional endpoints, including p16 and p21 expression, senescence-associated β-galactosidase (SA-β-gal) activity, osteogenic differentiation and adipogenic drift, to distinguish enhanced autophagy initiation from substrate accumulation caused by blocked terminal degradation [26,129].

### 4.2. Testing MLCS-Associated Coordination and Delivery as Intervention Nodes

MLCS are experimentally compelling candidate nodes, but their therapeutic tractability in MSC aging remains limited. Because contacts are nanoscale, transient and heterogeneous, colocalization alone cannot demonstrate functional restoration. Live-cell measurements of contact frequency, dwell time and spatial distribution should be linked causally to mitochondrial-cargo delivery, autophagosome–lysosome fusion, lysosomal acidification and proteolysis [38,39,68,74]. More broadly, selective pharmacological control of small GTPases remains challenging [130]. Whether this challenge can be overcome for Rab7 cycling or MLCS dynamics remains unknown.

MLCS-targeted intervention should therefore be treated as proof of concept rather than a mature pharmacological strategy. Constitutively active Rab7 Q67L prolongs mitochondria–lysosome contacts and alters contact–fission coupling in classical cell models [41]. Engineered tethering restored contact-dependent lysosomal acidification and autophagic flux in senescent human cells [39], providing a cross-model experimental precedent. In MSCs, apoptotic vesicles improve selected senescence phenotypes and alleviate bone loss through Rab7-dependent autolysosome formation [72], but do not demonstrate direct control of MLCS assembly. Any MSC-directed intervention must restore mitochondrial-cargo degradation and function, not merely increase contact abundance.

### 4.3. Enhancing Terminal Degradative Capacity: Rebuilding Lysosomal Degradation and Transcriptional Adaptation

As discussed in Section 3.3, insufficient terminal lysosomal degradation may represent one of the most actionable rate-limiting steps in MLQF mismatch. The key issue at this level is to determine which strategies restore acidification, hydrolysis and autophagy-lysosome gene programs, rather than simply showing that lysosomes are important [78,80]. TFEB activation can promote lysosomal biogenesis and the autophagy–lysosome transcriptional program [131]. In BMSCs from middle-aged mice and corresponding bone-aging models, CXM102-mediated TFEB nuclear translocation provides the most direct MSC-relevant evidence [80]. Separately, elevation of Tfeb in osteoblast-lineage cells increases bone mass and strength, providing bone-lineage rather than BMSC-specific support [132]. Other autophagy-lysosome interventions in mesenchymal-lineage cells provide useful context but should not be interpreted as direct evidence of TFEB-dependent MLQF restoration [133]. Whether TFEB activation improves terminal degradation of mitochondrial cargo still requires mitochondrial flux reporters and lysosomal blockade-based validation.

Lysosomal pH correction offers a complementary strategy. mTORC1 regulates luminal pH and proteolysis through reversible V-ATPase assembly [92,134]. In non-MSC models, restoration of lysosomal acidification can recover autophagy in metabolic disease [113], and lysosome-targeted acidic delivery can promote aggregate clearance [135]. These studies establish engineering feasibility, not efficacy in MSC aging. Cell selectivity, safety, mitochondrial-cargo flux and functional concordance require MSC-specific evaluation [78,131].

### 4.4. Multi-Node Coordination by Natural-Product-Derived Small Molecules and Endogenous Metabolites

Mixed-defect aging may combine high damage input, impaired delivery and low terminal capacity. Under these conditions, single-node activation may be insufficient when several defects coexist. Natural-product-derived molecules can modulate mitochondrial quality control, autophagy, lysosomal transcription and inflammation across models [114,116,117], but their pleiotropy does not establish coordinated MLQF repair. They are better considered as candidate components of stratified combinations than as universal anti-aging agents.

Urolithin A (UA) illustrates the need to separate broad mitochondrial-health effects from MLQF repair. UA induces PINK1-dependent mitophagy in T cells [114] and TFEB-related lysosomal programs in tumor-associated macrophages [116], but these are cross-model precedents. In human adipose-derived MSCs, UA attenuates chemotherapy-induced senescence, SASP and mitochondrial dysfunction [115]. Human supplementation studies report tolerability and improved mitochondrial-health indicators in middle-aged adults [136]. None of these studies directly demonstrates MLQF restoration in aged BMSCs.

Spermidine (SPD) inhibits histone acetyltransferase p300 (EP300) and promotes autophagy across models [117]. In human umbilical-cord MSCs, SPD delays replicative and hydrogen peroxide-induced senescence in association with SIRT3-dependent antioxidant signaling [118]. In lipopolysaccharide-stressed BMSCs, SPD is associated with metabolic, N6-methyladenosine and autophagy changes and improved osteogenic differentiation [119]. These studies provide MSC functional evidence, but do not identify the dominant MLQF bottleneck without mitochondrial flux assays.

**Figure 3:**
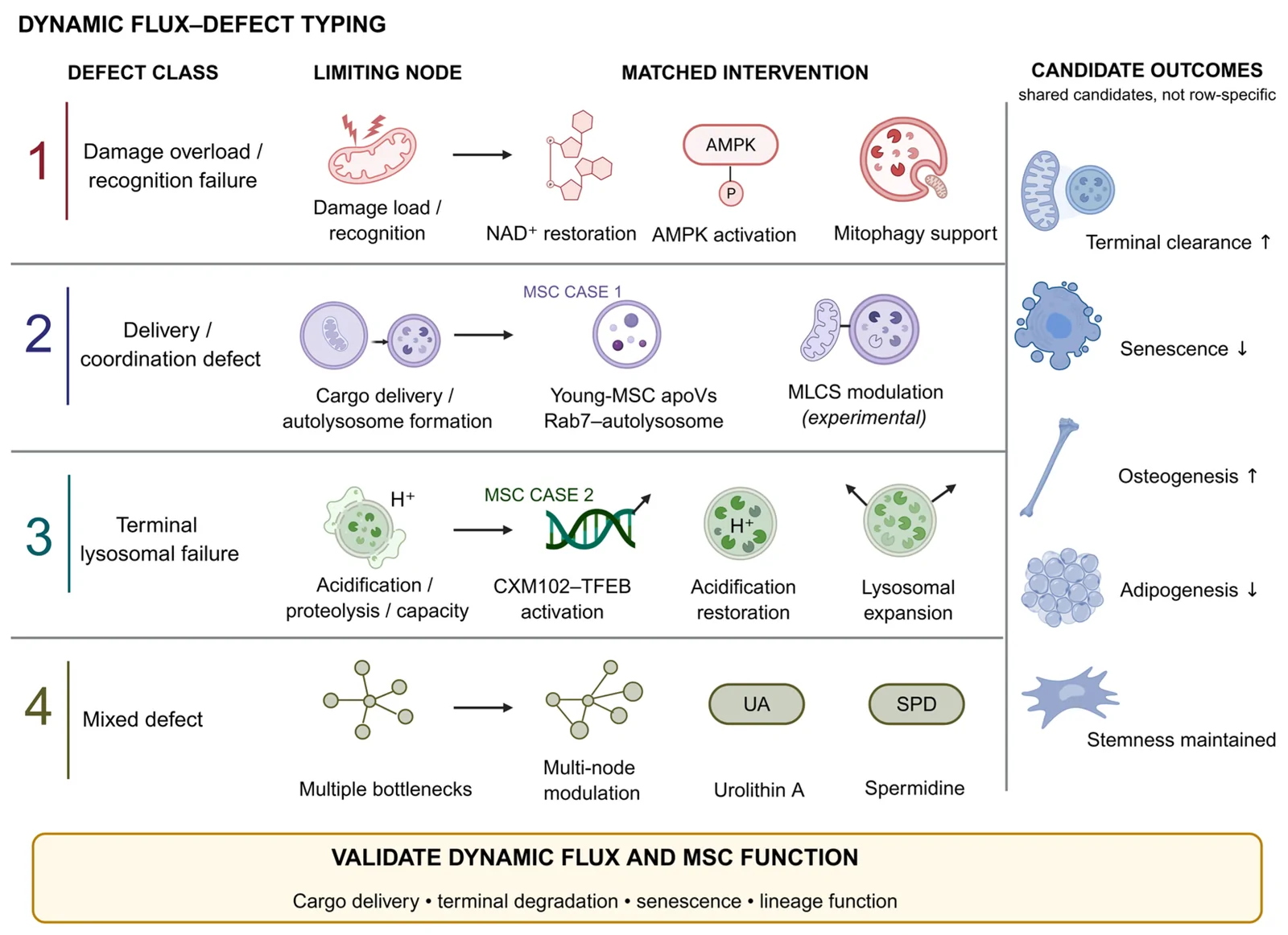
Bottleneck-guided intervention and validation workflow for MLQF defects in aging MSCs. Dynamic flux-defect typing distinguishes damage-input overload or impaired recognition, delivery-coordination defects, terminal lysosomal degradation failure, and mixed defects. Candidate interventions are matched to the dominant bottleneck, with strategies including NAD^+^- or AMPK-dependent upstream correction, experimental Rab7- or MLCS-associated modulation, TFEB-dependent lysosomal biogenesis, restoration of lysosomal acidification, and multi-node intervention. Urolithin A and spermidine are positioned as candidates for mixed defects rather than as pathway-specific therapies. Young-MSC-derived apoptotic vesicles (apoVs)–Rab7 modulation and CXM102–TFEB activation are presented as illustrative MSC-relevant examples; neither alone establishes restoration of mitochondrial cargo-specific terminal degradation. Intervention efficacy should be established using dynamic mitochondrial flux assays, lysosomal functional readouts, and MSC-relevant endpoints. Functional benefit remains conditional on demonstrable restoration of terminal clearance. Numbers 1–4 denote the four defect classes: (1) damage overload or recognition failure, (2) delivery or coordination defect, (3) terminal lysosomal failure, and (4) mixed defect. Black rightward arrows link each identified limiting node to the corresponding matched intervention, outward arrows indicate lysosomal expansion, and ↑/↓ indicate the intended direction of change in candidate outcomes. Candidate outcomes are shared across intervention classes and are not specific to individual rows.

Natural products and endogenous metabolites should therefore be evaluated after flux-defect stratification. The dominant bottleneck should determine the appropriate combination of NAD^+^- or AMPK-based upstream correction, Rab7-dependent autolysosome formation or experimental MLCS-associated modulation, and TFEB- or lysosome-dependent terminal repair. Figure 3 summarizes the sequence from defect identification to targeted intervention, flux reassessment and functional recovery.

## 5. Future Perspectives: From Framework Integration to Causal Validation

The translational value of MLQF lies in its analytical structure rather than in proposing a new molecular pathway. As an author-defined, evidence-graded framework, it places damage input, cargo delivery and terminal lysosomal degradation in one measurable chain and distinguishes driver nodes from amplifiers and correlates. Translation should therefore begin with identification of the flux-limiting bottleneck, followed by causal repair and reassessment of both mitochondrial-cargo degradation and MSC function. Future work should establish causal hierarchy, resolve heterogeneity across MSC sources and states, and convert validated mechanisms into stratification and intervention tools.

### 5.1. Clarifying Causal Hierarchy: From Association Maps to Actionable Nodes

Most MSC evidence currently shows association or improvement after intervention. Causality requires complementary loss- and gain-of-function experiments. Candidate nodes such as Rab7 cycling, MLCS dynamics and TFEB regulation should be perturbed in young MSCs to test whether they induce flux decline and premature senescence [43,137], with CRISPR screens used to nominate rather than validate drivers [138]. The dominant bottleneck should then be repaired in aged MSCs—for example, by restoring lysosomal biogenesis, acidification or Rab7-dependent autolysosome formation—and flux, SASP and differentiation assessed in parallel [72,80]. For contact-dependent acidification, the cross-model rescue achieved with engineered tethering [39] provides an experimental precedent, not evidence of MSC efficacy. A target is actionable only when node perturbation, flux change and phenotype are connected in the same MSC experiment.

Technically, dynamic flux reporter systems and high-throughput functional screening need to be integrated into a shared validation framework. Genetically encoded reporters such as mito-Keima have been used to analyze mitophagy flux in difficult-to-transfect primary cells, including pancreatic β cells, and in complex mouse models, providing methodological reference points for MSC studies. However, their application in primary MSCs and in vivo MSC models still requires optimization [139]. At the same time, genome-wide clustered regularly interspaced short palindromic repeat (CRISPR) interference (CRISPRi) screening in primary human MSCs can be used to systematically identify regulatory nodes involved in replicative and inflammatory senescence [137]. Integrating such screening results with mito-Keima or mito-QC readouts, lysosomal acidification measurements, BafA1 difference assays and MSC functional endpoints would help avoid overinterpretation of static markers and reduce candidate mechanisms to testable causal chains. Integrating such screening results with mito-Keima or mito-QC readouts, lysosomal acidification measurements, BafA1 difference assays and MSC functional endpoints would help avoid overinterpretation of static markers and reduce candidate mechanisms to testable causal chains (Box 1).

Box 1. **Testable predictions for validating the MLQF framework in MSC aging.**

**   1. Coordination-node disruption prediction**

   If Rab7 cycling, MLCS dissociation and reassembly, or TFEB-mediated lysosomal regulation is required to maintain MLQF, then selective impairment of the relevant node in young MSCs should reduce lysosome-dependent mitochondrial cargo turnover, even in the absence of a marked increase in mitochondrial damage input. Evidence for this prediction should combine mito-Keima or mito-QC measurements of acidic-compartment delivery with lysosomal-blockade experiments, mitochondrial cargo persistence, lysosomal pH or proteolytic activity, and MSC functional endpoints. The phenotype should include increased p21 and p16 expression, a higher proportion of SA-β-gal-positive cells and reduced osteogenic differentiation potential.

**   2. Upstream initiation is insufficient to rescue blocked terminal degradation**

   If the dominant bottleneck in aged MSCs lies at the level of terminal lysosomal degradation, then enhancing upstream autophagy initiation without restoring acidification, fusion or hydrolytic capacity should increase LC3-positive structures or autophagosome numbers but fail to increase completed mitochondrial cargo degradation or restore MSC function. Because mito-Keima or mito-QC signals may persist or accumulate when acidic-compartment delivery is uncoupled from degradation, these signals should be interpreted together with BafA1-sensitive cargo accumulation, mitochondrial cargo persistence, lysosomal pH and proteolytic activity.

**   3. Bottleneck-repair prediction**

  If insufficient lysosomal acidification or restricted Rab7-dependent autolysosome formation represents the key bottleneck, then targeted repair of this step should improve terminal degradation of damaged mitochondrial components, reduce SASP levels, restore osteogenic competence and restrain adipogenic drift in parallel.

**   4. Alternative-route compensation prediction**

   If MDVs or microautophagy-like delivery serve as compensatory routes when canonical mitophagy is restricted, then enhanced alternative delivery should become detectable after MSC-compatible reporter systems for MDV-mediated or microautophagy-like delivery of damaged mitochondrial components have been established. Once these tools have been validated, co-inhibition of the alternative route should be tested to determine whether it amplifies senescence-associated phenotypes.

### 5.2. Resolving Spatiotemporal Network Heterogeneity: Single-Cell Resolution and Microenvironmental Interactions

MSC heterogeneity poses a major challenge for flux-defect stratification and matched intervention [140,141,142]. MSCs from different tissue sources, subpopulations or microenvironmental stress states may rely on distinct rate-limiting steps within the quality-flux network. Integrating single-cell multi-omics with dynamic flux reporters could help generate a flux-state map of MSCs and identify candidate subpopulations in which quality-control imbalance emerges early. Such maps should move beyond cell-state annotation by linking damaged mitochondrial cargo delivery, terminal lysosomal degradation and MSC functional phenotypes. Disease-oriented multi-omic studies have begun to connect senescent skeletal stem/progenitor populations with osteoarthritis-relevant tissue remodeling, supporting the need to anchor single-cell maps to functional and pathological outputs [143].

Systemic inflammation, altered metabolites and matrix stiffening may reshape MSC quality control within the aging niche. Single-cell and spatial maps of human bone marrow, including ligand–receptor analyses, associate microenvironmental cues with mesenchymal or stromal-state changes [144,145]. Three-dimensional co-culture, organoid and tissue-engineering systems can test how immune, endothelial and mechanical signals alter flux. Their value will depend on coupling spatial context to dynamic MLQF readouts and actionable biomarkers.

### 5.3. Towards Stratified Monitoring and Mechanism-Guided Intervention: Translational Applications of the Flux Framework

Translation requires measurable readouts and clear cell-of-origin boundaries. Extracellular vesicles (EVs) isolated from frail individuals contain higher levels of mitochondrial DNA (mtDNA) and inflammatory proteins [146]. MSC-derived EVs may contain functional mitochondria or mitochondrial cargo [147]. In an osteoarthritis-related experimental setting, mitochondria-rich EVs from human synovial fluid-derived MSC cultures have been tested for their effects on stressed chondrocytes and disease progression [148]. These observations provide plausibility but do not show that circulating EV cargo reports BMSC MLQF. Cell-of-origin tracing, marrow sampling or MSC-specific labeling is required. Activatable imaging probes may complement this approach in organoid or in vivo models if cell specificity and validity are established.

Two therapeutic routes are plausible: intracellular repair through bone-targeted nanoparticles, liposomes, engineered EVs or ex vivo preconditioning; and niche remodeling through senescent-cell clearance, anti-inflammatory treatment or metabolic modulation. Bone-targeted clearance of marrow senescent cells improves osteogenic function and bone-mass parameters in age-related bone-loss models, supporting the feasibility of combining niche remodeling with targeted delivery [149]. This evidence does not yet establish MLQF-mediated efficacy.

## 6. Conclusions

MLQF is an author-proposed, evidence-graded framework that recasts MSC aging as a measurable mismatch between mitochondrial damage and lysosomal clearance. Its conceptual contribution is not the discovery of a new molecular pathway or the designation of MLCS as a universal hub. Rather, it separates acidic-compartment delivery from terminal degradation, grades direct MSC evidence against cross-model mechanisms and identifies the rate-limiting step before selecting single-node, sequential or combined interventions. Recent evidence that mitochondria–lysosome contacts support lysosomal acidification strengthens the framework’s biological plausibility, but the mechanism and its limiting role remain unverified in naturally aged MSCs. Dynamic flux assays should therefore be integrated with inflammatory and lineage endpoints, and targeted repair should be required to restore both mitochondrial-cargo degradation and MSC function.

## Figures and Tables

**Table 1:** Assays for mitochondrial delivery and degradation in MSCs.

Assay/System	Primary Readout	Supported Interpretation	Key Interpretative Boundary	References
mito-Keima	pH-dependent red-shifted mitochondrial signal	Delivery of mitochondrial cargo to acidic compartments	Requires optimized expression and imaging or flow settings in MSCs; does not alone quantify terminal lysosomal degradation	[23,26]
LC3-II + BafA1	LC3-II, p62 or mitochondrial cargo accumulation after lysosomal blockade	General autophagic flux; auxiliary mitophagy assessment when paired with cargo markers	Not mitochondria-specific without mitochondrial cargo readouts	[22,51]
mito-QC	GFP quenching with mCherry retention after acidic delivery	Visualization of mitochondrial delivery to acidic compartments in vitro or in vivo	Requires stable expression or transgenic systems; signal should be interpreted with degradation-capacity assays	[27,53]
Electron microscopy with immunogold labeling	Ultrastructural localization of mitochondria and lysosomal compartments	Structural evidence for cargo localization and route assignment	Static snapshot; not a standalone flux or degradation assay	[37,54]

**Table 2:** Lysosome-directed routes of mitochondrial quality control.

Route	Lysosomal Delivery Logic	Key Machinery	Evidence Status and Context in MSCs	References
Mitophagy	Autophagosome-mediated engulfment of whole mitochondria or large mitochondrial portions	PINK1/Parkin-associated machinery, LC3-II and ATG machinery	Supported by MSC functional and flux-related evidence; clears whole mitochondria or mitochondrial fragments.	[46,47,48]
MDVs	Local mitochondrial budding enables component-level delivery to endosomes or lysosomes	TOLLIP, Parkin and Rab GTPases	Candidate route; direct MSC flux evidence is limited; may remove selected cargo from locally damaged regions.	[57,59]
Microautophagy-like pathway	Direct invagination of lysosomal or late endosomal membranes enables uptake of cytoplasmic material, including mitochondrial components	ESCRT complexes, ATG machinery and lysosomal membrane proteins	Candidate route supported mainly by non-MSC models; may mediate piecemeal mitochondrial cargo clearance.	[54,65]

**Table 4:** Intervention nodes across the MLQF axis.

Bottleneck	Strategy/Target	Main Role	Evidence Level	Key Boundary	References
Upstream damage sensing	NAD^+^ supplementation or NAD^+^ synthesis/salvage strategies, represented by NMN	Redox and metabolic correction	MSC and skeletal-context evidence	Not direct proof of restored terminal degradation	[100,107,108]
Upstream damage sensing	AMPK activators, such as AICAR and metformin	Energy sensing and autophagy initiation	MSC functional and autophagy-marker evidence	Mitochondria-specific flux validation remains needed	[109,110,111,112]
MLCS/delivery coordination	Rab7 restoration or supplementation via apoptotic vesicles	Autolysosome formation and cargo delivery	MSC vesicle and skeletal phenotype evidence	Supports Rab7-autolysosome axis, not direct MLCS manipulation	[72]
MLCS/delivery coordination	Matrix-mechanics modulation	MLCS and mitochondrial network remodeling	Direct MSC evidence	Direct degradation-flux evidence remains limited	[43]
Terminal degradation	TFEB-related activation, represented by CXM102	Lysosomal biogenesis and autophagy-lysosome program	MSC and bone-aging evidence	Mitochondrial-cargo flux validation remains needed	[80]
Terminal degradation	Lysosomal acidification or V-ATPase-related regulation	Low-pH restoration and hydrolytic capacity	Stronger non-MSC evidence; indirect MSC support	Closed-loop MSC flux validation remains insufficient	[77,78,92,113]
Mixed defects	Urolithin A	Mitophagy and possible lysosomal adaptation	MSC stress-model evidence plus cross-model mechanisms	TFEB-related mechanism is not yet MSC-specific	[114,115,116]
Mixed defects	Spermidine	EP300 inhibition, autophagy and antioxidant regulation	Relatively direct MSC functional evidence	Dominant MLQF node remains undefined	[117,118,119]

Note: Evidence categories distinguish direct MSC findings, in vivo skeletal phenotypes and cross-model mechanistic support. Neither an in vivo skeletal phenotype nor a cross-model mechanism alone establishes causal modulation of MLQF in MSCs.

## Data Availability

No new data were created or analyzed in this study. Data sharing is not applicable to this article.

## Abbreviations

The following abbreviations are used in this manuscript:

AMPK	AMP-activated protein kinase
BafA1	bafilomycin A1
BMSC	bone marrow-derived mesenchymal stem/stromal cell
cGAS	cyclic GMP–AMP synthase
ESCRT	endosomal sorting complex required for transport
LC3	microtubule-associated protein 1 light chain 3
MDV	mitochondria-derived vesicle
MLCS	mitochondria–lysosome contact site
MLQF	mitochondria–lysosome quality flux
SASP	senescence-associated secretory phenotype
STING	stimulator of interferon genes
TFEB	transcription factor EB
